# Alkaloids from *Fissistigma latifolium* (Dunal) Merr

**DOI:** 10.3390/molecules15074583

**Published:** 2010-06-24

**Authors:** Asmah Alias, Hazrina Hazni, Faridahanim Mohd Jaafar, Khalijah Awang, Nor Hadiani Ismail

**Affiliations:** 1 Faculty of Applied Sciences, Universiti Teknologi Mara, 40450 Shah Alam, Selangor, Malaysia; 2 Department of Chemistry, Universiti Malaya, Kuala Lumpur, Malaysia

**Keywords:** *Fissistigma latifolium*, Annonaceae, alkaloids

## Abstract

A phytochemical study of the bark of *Fissistigma latifolium* (Annonaceae) yielded a new aporphine alkaloid, (-)-*N*-methylguattescidine (**1**), and eight known alkaloids: liriodenine (**2**), oxoxylopine (**3**), (-)-asimilobine (**4**), dimethyltryptamine (**5**), (-)-remerine (**6**), (-)-anonaine (**7**), columbamine (**8**) and lysicamine (**9**). The compounds were isolated using various chromatographic methods and structural elucidation was accomplished by means of spectroscopic methods, notably 1D-NMR (^1^H, ^13^C, DEPT), 2D-NMR (COSY, HMQC, HMBC), UV, IR and MS.

## 1. Introduction

*Fissistigma latifolium* (Dunal) Merr. from the genus *Fissistigma* is a climbing shrub found in low land forest of Malaysia, Sumatra, Borneo and Philippines [1]. The genus *Fissistigma* (Annonaceae) consists of about 80 species and is widely distributed in Asia and Australia [2]. Our previous studies on *F. fulgens* and *F. manubriatum* have resulted in the isolation of aporphine, oxoaporphine and protoberberine alkaloids [3,4]. In this paper, we report the isolation and characterization of a new aporphine alkaloid, (-)-*N*-methylguattescidine (**1**), from *F. latifolium*. This alkaloid, together with eight known alkaloids, namely liriodenine (**2**), oxoxylopine (**3**), (-)-asimilobine (**4**), dimethyltryptamine (**5**), (-)-remerine (**6**), (-)-anonaine (**7**), columbamine (**8**) and lysicamine (**9**), were obtained from the methanol extract of the bark of the plant.

**Figure 1:**
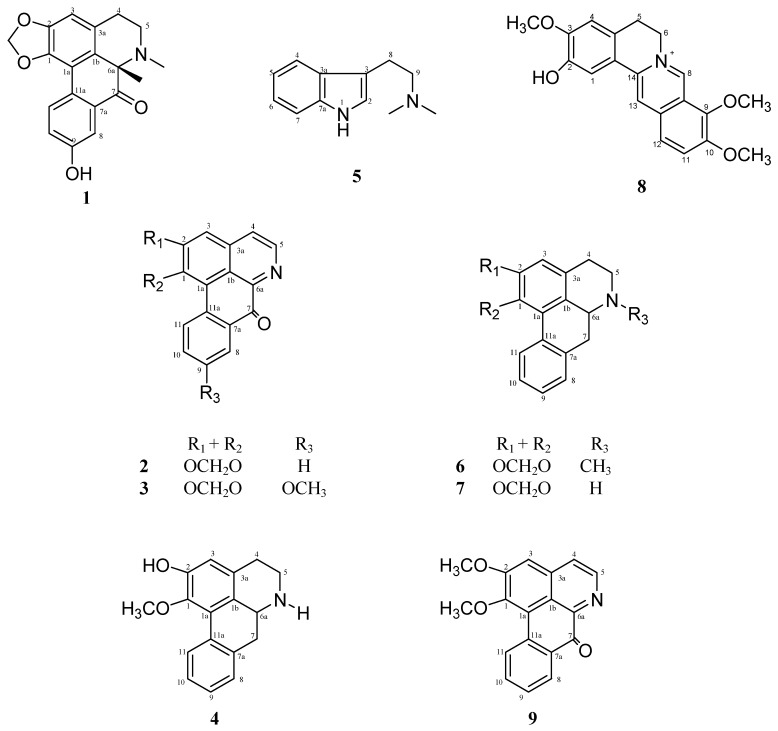
Alkaloids **1**-**9**, isolated from *Fissistigma latifolium.*

## 2. Results and Discussion

(-)-*N*-Methylguattescidine (**1**) exhibited a molecular formula of C_19_H_17_O_4_N based on the HRESIMS spectrum (positive mode), which showed a pseudomolecular ion at *m/z* 324.3581 [M+H]^+^ (calcd. 324.3595). The UV spectrum showed an absorption band at 310 nm, suggesting the compound was an aporphine alkaloid with substitutions at position 1 and 2 [5]. The IR spectrum indicated the presence of a C=O band at 1,710 and an OH one at 3,409 cm^-1^. The ^13^C-NMR spectrum showed presence of 19 carbons. The signal at δ 198.0 ppm confirmed the presence of the carbonyl group, while the signal at δ 153.1 ppm is evidence for the oxygenated aromatic carbon. The DEPT spectrum revealed three methylene carbons at δ 26.9 ppm, 41.4 ppm and 96.9 ppm. Signal at δ 96.9 ppm is indicative of a methylenedioxy carbon. This is consistent with two doublets at δ 5.99 ppm (*J* = 1.2 Hz) and δ 6.07 ppm (*J* = 1.2 Hz) in the ^1^H-NMR spectrum for the protons of methylenedioxy group which is typically located at positions 1 and 2. The characteristic ABD aromatic signals of H-11, H-10 and H-8 of aporphine alkaloid were observed at δ 8.24 ppm (*d, J = *8.7 Hz), δ 7.13 ppm (*dd, J = *8.7, 2.7 Hz) and δ 7.39 ppm (*d, J = *2.7 Hz), respectively. The singlet at δ 6.54 ppm is due to H-3 while two methylene signals at position 4 and 5 appeared as multiplets between δ 3.01–2.55 ppm. Assignment of C-4 at δ 26.9 ppm was made based on the HMQC correlations to H-4 (δ 2.55 ppm) and H-4’ (δ 3.00 ppm). Similarly, C-5 at δ 41.4 ppm showed correlation to H-5 (δ 2.99 ppm) and H-5’ (δ 3.01 ppm). The ^1^H-NMR spectrum also exhibited an *N*-methyl signal at δ 2.34 ppm and another methyl group attached to C-6a gave a singlet at δ 1.52 ppm. The assignment of this methyl group at the 6a position is confirmed through its HMBC correlation with C-6a at δ 62.7 ppm, C-1b at δ 118.3 ppm and C-7 at δ 198.0 ppm. The quaternary carbon signals were assigned based on HMBC experiment. C-1a at δ 108.9 ppm, C-7a at δ 126.0 ppm and C-9 at δ 153.1 ppm were assigned based on their correlations with H-11 at δ 8.24 ppm, while C-1b at δ 118.3 ppm and C-2 at δ 143.2 ppm showed correlations with H-3 at δ 6.54 ppm. Table 1 summarizes the ^1^H- and ^13^C-NMR data of compound **1**. 

**Table 1:** ^1^H-NMR (300 MHz) and ^13^C-NMR (75 MHz) spectral data of compound **1** in CD_3_OD (δ in ppm, *J* in Hz**).**

Position	^1^H-NMR (δ ppm)	^13^C-NMR (δ ppm)	HMBC
1	-	138.8	
1a	-	108.9	
1b	-	118.3	
2	-	143.2	
3	6.54, *s*	103.9	C-1b, C-1, C-2
3a	-	125.3	
4	2.55, 3.00, *m*	26.9	
5	2.99, 3.01, *m*	41.4	
6a	-	62.7	
7	-	198.0	
7a	-	126.0	
8	7.39, *d, (J = *2.7 Hz*)*	110.3	C-9, C-7
9	-	153.1	
10	7.13, *dd,( J_o_* = 8.7 Hz, *J_m_* = 2.7 Hz)	122.2	C-11a
11	8.24, d, (*J* = 8.7 Hz)	122.7	C-1a, C-7a, C-9
11a	3.52, *m*	123.1	
O-CH_2_-O	5.99, 6.07, *d, (J = *1.2 Hz*)*	96.9	C-1, C-2
N-CH_3_	2.34, *s*	34.1	C-6a
CH_3_	1.52, *s*	25.0	C-6a, C-1b, C-7

Based on all spectroscopic data compound **1** was identified as (-)-*N*-methylguattescidine, a new 6a-methylated-7-oxo-aporphine alkaloid. Occurrence of 6a-methylated aporphine alkaloids is very rare, having only been previously reported by Reynald *et al*. [6]. Apart from the N-CH_3_ signal at δ 2.34 ppm, the signals of all other protons in compound **1** are in a good agreement with that of guattescidine. The slight differences in the chemical shifts may be contributed to the different solvents used for the NMR measurement. The optical rotation of all known isolated compounds were found to be (-ve) thus corresponding to β configuration [7]. Therefore, based on biogenetic reasoning, the configuration at C-6a for (-)-*N*-methylguattescidine is deduced to be β.

Other isolated compounds from this plant are three aporphine alkaloids, (-)-asimilobine, (-)-anonaine and (-)-remerine; three oxoaporphine alkaloids, liriodenine, oxoxylopine and lysicamine; one tryptamine alkaloid, dimethyltryptamine and one protoberberine alkaloid, columbamine. These compounds were characterized based on analysis of spectroscopic data and comparison with literature data. 

## 3. Experimental

### 3.1. General

The ^1^H-NMR and ^13^C-NMR were recorded in CDCl_3_ and CD_3_OD on a Bruker 300 Ultrashield NMR spectrometer measured at 300 and 75 MHz. Chemical shifts are reported in ppm and δ scale and the coupling constants are given in Hz. Melting points were taken on a hot stage Gallen Kamp melting point apparatus with microscope and were uncorrected. The infrared (IR) was recorded on a Perkin Elmer spectrum one FT-IR spectrometer using CH_2_Cl_2_ as solvent. Optical rotations were measured on a JASCO P1020 digital polarimeter. HRESIMS was obtained on a Thermo Finnigan Automass Multi. The ultraviolet (UV) spectra were obtained in ethanol on a Shimadzu UV-Vis 160i instrument. 

### 3.2. Plant material

The bark of *Fissistigma latifolium *(Dunal) Merr. (KL 4623) was collected from Dungun, Terengganu and was identified by L.E. Teo (Department of Chemistry, University of Malaya) and all plant materials were screened for their alkaloidal content before any chemical analysis. Voucher specimens were deposited at the Herbarium, Department of Chemistry, University of Malaya.

### 3.3. Extraction and isolation of the alkaloids

Dried and ground bark of *F. latifolium* (1 kg) was defatted with petroleum ether (5 L) overnight before being extracted with dichloromethane (7.5 L) and methanol (10 L) for eight hours using a Soxhlet extractor. The methanol extract was subjected to acid-base extraction. The crude alkaloids (3.5 g) from the MeOH extract were subjected to SPE flash column using a gradient elution system of hexane (hex):ethyl acetate (EA) and dichloromethane (DCM):methanol (MeOH) to give 29 fractions. The combined fractions 19–20 were subjected to PTLC using a 95:5 DCM:MeOH solvent system to yield a mixture of compounds **2** (15.3 mg) and **3 **(7.0 mg) [8,9]. These compounds were separated by repeated PTLC using the same solvent system. Fraction 23 from the SPE column was subjected to PTLC using a 95:5 DCM:MeOH solvent system to obtain compounds **4 **(5.2 mg) and **5 **(5.7 mg) [10,11]. The combined fractions 26 and 27 from the SPE were subjected to column chromatography using a gradient elution system of hex:EA and DCM:MeOH to obtain 13 fractions. Fractions 7–10 were combined and subjected to PTLC using a 93:7 DCM:MeOH solvent system to yield compounds **6 **(6.4 mg) and **7 **(8.2 mg) [12,13]. The combined fractions 11–13 were subjected to PTLC using a 90:10 DCM:MeOH solvent system to obtain alkaloid **1 **(5.1 mg). Fraction 29 from the SPE was subjected to PTLC using a 90:10 DCM:MeOH solvent system to yield compounds **8** (4.6 mg) and **9 **(3.9 mg) [14,15]. Spectral data for compounds **2**-**9** were in agreement with published data [8,9,10,11,12,13,14,15]. 

*(-)-N-Methylguattescidine* (**1**). yellow amorphous solid; [α]^30^*_D_*: -20º (c = 0.1 mg mL^-1^, CHCl_3_); MS *m/z*: 324.1242, C_19_H_17_O_4_N; UV λ_max_ nm EtOH: 235, 310; IR υ_max_ cm^-1^: 3409, 1710, 1266; ^1^H-NMR and ^13^C NMR data, see Table 1.

## 4. Conclusions

The phytochemical study on the bark of *Fissistigma latifolium* (Annonaceae) yielded a new aporphine alkaloid, (-)-*N*-methylguattescidine (**1**), and the eight known alkaloids liriodenine (**2**), oxoxylopine (**3**), (-)-asimilobine (**4**), dimethyltryptamine (**5**), (-)-remerine (**6**), (-)-anonaine (**7**), columbamine (**8**) and lysicamine (**9**). Tryptamine alkaloids have never been reported from *Fissistigma* species, whereas the new compound (-)-*N*-methylguattescidine (**1**) represents a rare finding of a naturally occurring 6a-methylated-7-oxo-aporphine alkaloid.
